# Epidemiology of Lyme borreliosis through two surveillance systems: the national Sentinelles GP network and the national hospital discharge database, France, 2005 to 2016

**DOI:** 10.2807/1560-7917.ES.2019.24.11.1800134

**Published:** 2019-03-14

**Authors:** A Septfons, T Goronflot, B Jaulhac, V Roussel, S De Martino, S Guerreiro, T Launay, L Fournier, H De Valk, J Figoni, T Blanchon, E Couturier

**Affiliations:** 1 Santé publique France, Paris, France; 2 European Programme for Intervention Epidemiology Training (EPIET), European Centre for Disease Prevention and Control (ECDC), Stockholm, Sweden.; 3 Sorbonne Université, INSERM, Institut Pierre Louis d’Epidémiologie et de Santé Publique IPLESP, AP-HP, Hôpital Saint Antoine, Paris, France; 4 Early Bacterial Virulence: Lyme borreliosis Group, Université de Strasbourg, CHRU Strasbourg, Fédération de Médecine Translationnelle de Strasbourg, VBP EA 7290, Strasbourg, France; 5 Centre National de Référence des *Borrelia*, Hôpitaux Universitaires de Strasbourg, Strasbourg, France

**Keywords:** Lyme borreliosis, neuroborreliosis, general practitioners, hospitalisation, incidence, surveillance

## Abstract

Background: Lyme borreliosis (LB) is the most frequent vector-borne disease in France. Since 2009, surveillance of LB is conducted by a sentinel network of general practitioners (GPs). This system, in conjunction with the national hospitalisation database was used to estimate the incidence and describe the characteristics of LB in France.

Aim: To describe the estimated incidence and trends in GP consultations and hospital admissions for LB in France and identify risk groups and high-incidence regions.

Results: From 2011 to 2016, the mean yearly incidence rate of LB cases was 53 per 100,000 inhabitants (95% CI: 41–65) ranging from 41 in 2011 to 84 per 100 000 in 2016. A mean of 799 cases per year were hospitalised with LB associated diagnoses 2005–16. The hospitalisation incidence rate (HIR) ranged from 1.1 cases per 100,000 inhabitants in 2005 to 1.5 in 2011 with no statistically significant trend. We observed seasonality with a peak during the summer, important inter-regional variations and a bimodal age distribution in LB incidence and HIR with higher incidence between 5 and 9 year olds and those aged 60 years. Erythema migrans affected 633/667 (95%) of the patients at primary care level. Among hospitalised cases, the most common manifestation was neuroborreliosis 4,906/9,594 (51%).

Conclusion: Public health strategies should focus on high-incidence age groups and regions during the months with the highest incidences and should emphasise prevention measures such as regular tick checks after exposure and prompt removal to avoid infection.

## Introduction

Lyme borreliosis (LB) is caused by spirochaetes of the *Borrelia burgdorferi* sensu lato species complex, which are transmitted by different *Ixodes* spp. ticks [1]. The most common clinical manifestation is erythema migrans (EM). However, in the absence of antibiotic treatment the infection can spread and cause severe manifestations affecting a patient's skin, nervous system, joints, or heart [1].

LB is the most common tick-borne infectious disease in North America [2,3] and countries with temperate climates within Europe and Asia [4,5]. Incidence of LB has been increasing in some European countries [6-12] and it has been suggested that LB will become a more prominent health concern with predicted climate changes potentially impacting tick density and geographical distribution [4]. Therefore, knowledge of epidemiological characteristics of LB is important to decide on allocation of resources and to target prevention measures [13].

 Since 2009, Lyme disease has been monitored by the general practitioners of the Sentinelles network. This network is a sentinel network of general practitioners (GPs) and operates as routine, systematic and standardised surveillance system allowing for trend analyses of LB incidence and the estimation of national and regional LB incidence rates. Between 2009 and 2012, the national estimated incidence rate was stable [14]. Between 2001 and 2012, LB incidence estimated by regional studies varied considerably on a regional level from 24 cases per 100,000 inhabitants in Aquitaine (south-western France) to 232 cases per 100,000 inhabitants in Alsace (eastern France) [15]. Another source of LB data in France is the national hospital discharge base. Between 2004 and 2009, the average annual LB-associated hospitalisation rate was estimated as 1.55 cases per 100,000 inhabitants; important regional variations in hospitalisation rates were also observed [14].

In the present study, we estimated the annual incidence of LB cases diagnosed at primary care level between 2011 and 2016 in mainland France and describe the characteristics of these cases. We also estimated the incidence of hospitalised LB cases between 2005 and 2016, with a particular focus on Lyme neuroborreliosis (LNB).

## Methods

### The Sentinelles network

The Sentinelles network, established in 1984 is a real-time epidemiologic surveillance system comprised of a sample of GPs located throughout mainland France, who participate on a voluntary basis [14,16,17]. The sentinel general practitioners (SGPs) report new LB diagnoses on a weekly basis as part of the Sentinelles surveillance system since 2009. A comparison between SGPs and GPs found that they are similar in terms of age, but SGPs have slightly more consultations per week; the impact of this difference on incidence estimates is small [17].

All reported LB cases were validated by an expert group constituted by clinicians, microbiologists and epidemiologists applying the European Union Concerted Action on Lyme Borreliosis (EUCALB) case definitions (Box 1) [18].

Box 1 **Sentinelles network case definition for Lyme borreliosis**

(i) Presence of EM

(ii) Arthritis, cutaneous (other than EM) or heart manifestations associated with LB confirmed by ELISA and Western blot, or

(iii) Neurological manifestations associated with LB confirmed by ELISA and Western blot, associated with the presence of antibodies in the cerebrospinal fluid (CSF)

In the presence of meningoradiculitis or unilateral facial paralysis, cases were validated even in the absence of CSF fluid analysis, if clinically suggestive (consensus agreement) in patients with serological confirmation (ELISA and Western blot) and who reported a history of EM less than 2 months before the onset of neurological manifestations.

EM: Erythema migrans.

Information was collected from cases by the SGP during the medical consultation using a standardised questionnaire developed by the Sentinelles network (Box 2).

Box 2 **Information collected from standardised questionnaire, Sentinelles network, France, 2009–2016**

• Age

• Sex

• Date of diagnosis

• History and dates of tick bites

• Presence and description of EM, i.e. solitary or multiple, central clearing, centrifugal expansion and, since 2011, diameter in centimetre

• Other cutaneous manifestations, i.e. cutaneous lymphocytoma, acrodermatitis chronica atrophicans

• Arthritis (affected joint(s))

• Cardiac manifestations such as atrioventricular block, pericarditis, myocarditis, other

• Presence of neurological manifestations such as meningoradiculitis, clinical signs of meningitis, meningoencephalitis, radiculitis, facial paralysis, events related to another cranial nerve

• Date and results of serological tests and/or CSF analyses and reason(s) for hospitalisation

CSF: cerebrospinal fluid; EM: Erythema migrans.

### Data analysis

Data on LB cases reported to the Sentinelles network 1 January 2011–31 December 2016 were analysed. Estimated LB incidence rates were calculated as follows: the average number of cases notified by SGPs (adjusted for participation and geographic distribution) multiplied by the total number of GPs practicing in France (or in a given region for regional incidence rates) [17] divided by the total French population [19]. Confidence intervals (CI) were estimated under the assumption that the number of reported cases followed a Poisson distribution. In addition, we estimated annual incidence rates for the following four subgroups of cases: EM regardless of diameter, EM greater or equal to 5 cm, early and late disseminated LB and all cases except EM smaller than 5 cm (corresponding to EM ≥ 5 cm or disseminated LB).

### Lyme borreliosis hospitalisations, 2005–2016

The French national hospital discharge database (Programme de Médicalisation des Systèmes d’Information – PMSI) collects information on every hospital stay in France [20]. Each hospital discharge report, corresponding to a hospital stay, is described according to the following items: reasons of hospitalisation (principal diagnosis) and related medical conditions (associated diagnoses) coded with the tenth revision of the International Classification of Diseases (ICD-10) [21], length of hospital stay and characteristics of the patient (age, sex, place of hospitalisation and residence). A unique patient identifier allows the identification of multiple hospital stays for the same patient.

Hospital discharge reports with a principal or associated diagnosis of LB and admitted to hospital in France 1 January 2005–31 December 2016 were extracted from the PMSI. LB diagnoses were identified using the ICD10 codes: A69.2 for Lyme disease, M01.2 for arthritis in Lyme disease and L90.4 for acrodermatitis chronica atrophicans (ACA).

As described in the previous study [14] and due to the poor predictive value of the LB codes in the PMSI [22], we included discharge reports which met the following criteria: (i) a LB specific diagnosis (M01.2 or L90.4); (ii) a A69.2 code in the absence of any other diagnosis, or (iii) a A69.2 code associated with code(s) compatible with LB symptoms (neurological, cardiac, articular and ocular disorders) (Table 1). Hospital discharge reports with no patient identifier and those of patients living outside mainland France were excluded.

**Table 1:** ICD-10 codes of clinical disorders that may be related to Lyme borreliosis

Chapter VI: Diseases of the nervous system	ICD-10 code concerned
Meningitis	G00, G00.9, G01, G02, G03,G03.0, G03.1, G03.8, G03.9
Encephalitis, myelitis and encephalomyelitis	G04, G04.2, G04.8, G04.9, G05, G05.0, G05.2, G05.8
Disorders of trigeminal nerve	G50.8, G50.9
Facial nerve disorders	G51, G51.0,G51.8, G51.9
Disorders of other cranial nerves	G52, G52.0–3, G52.7–9
Cranial nerve disorders in diseases classified elsewhere	G53, G53.1, G53.8
Nerve root and plexus disorders	G54, G54.0–5, G54.8–9
Other polyneuropathies	G62, G62.8–9
Polyneuropathy in diseases classified elsewhere	G63, G63.0
Other disorders of peripheral nervous system	G64
**Chapter VII: Diseases of the eye and adnexa**	
Iridocyclitis	H20, H20.0–1, H20.8–9
Other disorders of iris and ciliary body	H21, H21.8–9
Disorders of iris and ciliary body in diseases classified elsewhere	H22, H22.0, H22.1, H22.8
Chorioretinal inflammation	H30, H30.0–9
Other disorders of choroid	H31, H31.8–9
Chorioretinal disorders in diseases classified elsewhere	H32, H32.0, H32.08, H32.8
**Chapter IX: Diseases of the circulatory system**	
Acute pericarditis	I30, I30.0–9
Pericarditis in diseases classified elsewhere	I32, I32.0–8
Acute myocarditis	I40, I40.0–9
Myocarditis in diseases classified elsewhere	I41, I41.0, I41.2, I41.8
Cardiomyopathy	I42, I42.9
Cardiomyopathy in diseases classified elsewhere	I43, I43.0
Atrioventricular and left bundle-branch block	I44, I44.0–7
Other conduction disorders	I45, I45.0–5, I45.8–9
Other heart disorders in diseases classified elsewhere	I52, I52.0–8
**Chapter XIII: Diseases of the musculoskeletal system and connective tissue**	
Arthritis and polyarthritis due to other specified bacterial agents	M00.8, M00.80–9
Direct infections of joint in infectious and parasitic diseases classified elsewhere^a^	M01, M01.30–9, M01.80–9
Other arthritis	M13, M13.0–9
Arthropathies in other diseases classified elsewhere	M14, M148

ICD10: tenth revision of the International Classification of Diseases.

^a^ Other than M01.20–9 codes: Arthritis in Lyme disease.

ICD-10 codes identified from [21].

We defined a case of LB as a person hospitalised in mainland France for LB (following the criteria above) at the first stay during the time period 2005–16. We defined a case of LNB as a person hospitalised with at least one of the specific neurological disorders listed in Table 1, associated with a LB disease code. We described the annual number of cases (patients) and the number of hospital stays and geographical distribution of patients according to place of residence, or if unknown, place of hospitalisation.

In a given geographical area, the hospitalisation incidence rate (HIR) was calculated by dividing the number of hospitalised cases (excluding repeat admissions) observed in the defined time period by the number of inhabitants of the geographical area (estimated by the National Institute of Statistic’s and Economic Studies [19] and then multiplied by 100,000. We also estimated hospitalisation rates per age group. To assess statistically significant changes in hospitalisation rates over the study period we performed a negative binomial regression with the number of cases per year as a dependent variable and annual population when hospitalisation occurred as exposure.

## Results

### The Sentinelles network (2011–2016)

The total number of SGPs that participated between 2011 and 2016 was 723, which is 1.17% of the total number of GPs in France in 2016 (n = 61,789). The number of SGPs who participated varied year to year, with the lowest number participating in 2013 and the highest in 2015 (332 and 455, respectively).

Over the study period, 932 LB cases were reported by SGPs of which, 265 were excluded by the expert group for the following reasons: did not meet the case definition (n = 61), clinical manifestation not described (n = 146), absence of serology confirmation for disseminated LB (n = 44), no lumbar puncture for neurological manifestations, except for meningoradiculitis or unilateral facial paralysis (n = 14). A total of 667 LB cases were included (94 in 2011, 85 in 2012, 113 in 2013, 76 in 2014, 105 in 2015 and 194 in 2016).

Among LB cases, 340 (53%) were female ranging from 46% to 57% depending on the year (Table 2). The median age was 54 years.

**Table 2:** Demographic and clinical characteristics of Lyme borreliosis cases, Sentinelles Network, mainland France, 2011–2016 (n = 667)

Characteristic	N	%	Median (range)
**Sex (md = 23) **			
Female	340	53	
**Male**	304	47	
**Age**	NA	NA	54 (1–91)
**Clinical characteristics**			
Erythema migrans^a^	633	95	
≥ 5cm (md = 14)	465	75	
Solitary lesion (md = 16)	591	96	
Central clearing (md = 52)	393	68	
Centrifugal extension (md = 61)	537	94	
Disseminated Lyme borreliosis	34	5	
Acrodermatitis	6	17	
Lymphocytoma	4	12	
Arthritis	17	50	
Radiculitis	4	12	
Facial paralysis	2	6	
Radiculitis and meningoradiculitis	1	3	
**Tick exposure**			
Tick bite (md = 83)	414	71	
Days between bite and diagnosis (md = 93)	NA	NA	11 (1–250)
In mainland France (md = 9)	403	99	
Hospitalisation (md = 103)	4	1	

md: missing data; NA: not applicable.

^a^ For individuals with Erythema migrans multiple clinical characteristics could be reported.

Of 667 diagnoses, 633 were EM diagnoses (95%), 591 (96%) were solitary lesions, 465 (75%) were equal or greater than 5 cm, 537 (94%) had a centrifugal extension and 393 (68%) had a central clearing (Table 2). A total of 34 cases presented with disseminated LB (5%), corresponding to arthritis (n = 17), acrodermatitis (n = 6), lymphocytoma (n = 4), radiculitis (n = 4), facial paralysis (n = 2) and both meningoradiculitis and radiculitis (n = 1).

A tick bite was reported in 414 cases (71%), among which 403 (97%) were diagnosed with EM. The median time between tick bite and diagnosis for the 414 cases was 10 days for all LB cases (range 1–250) (Table 2).

The estimated annual incidence rate of LB over the period 2011–16 averaged 53 cases per 100,000 inhabitants (95% CI: 41–65). This rate varied between 41 cases per 100,000 in 2011 to 84 in 2016, when the incidence increased significantly compared with previous years (Table 3). The estimated average annual incidence rate per 100,000 inhabitants was 50 cases (95% CI: 38–62) for EM (regardless of size), 37 cases (95% CI: 27–47) for EM ≥ 5 cm, three cases (95% CI: 0–5) for disseminated LB and 40 (95% CI: 30–51) for EM ≥ 5 cm or disseminated LB (Table 3). In 2016, only LB cases diagnosed with EM increased significantly, as opposed to cases diagnosed with disseminated LB.

**Table 3:** Yearly incidence rates of Lyme borreliosis by clinical manifestation, in general medicine, Sentinelles network, mainland France, 2011–2016

Cases	Incidence rate per 100,000 inhabitants (95% CI)						
	2011	2012	2013	2014	2015	2016	Average
Number of cases included	94	85	113	76	105	194	NR
Lyme borreliosis	41 (31–51)	44 (32–56)	55 (43–67)	41 (30–52)	51 (38–64)	84 (70–98)	53 (41–65)
Erythema migrans^a^	37 (27–46)	41 (29–53)	54 (42–66)	39 (29–49)	50 (38–63)	80 (66–93)	50 (38–62)
Erythema migrans ≥ 5 cm	30 (22–38)	28 (18–38)	45 (35–56)	25 (16–33)	37 (25–48)	59 (47–71)	37 (27–47)
Disseminated Lyme borreliosis	5 (1–8)	3 (0–6)	1 (0–3)	2 (0–4)	1 (0–3)	4 (1–7)	3 (0–5)
Erythema migrans ≥ 5 cm or disseminated Lyme borreliosis	35 (26–44)	31 (21–41)	47 (36–58)	26 (18–35)	38 (26–49)	63 (51–75)	40 (30–51)

CI: confidence interval; NR: not reported.

^a^ Regardless of the size.

**Figure 1:**
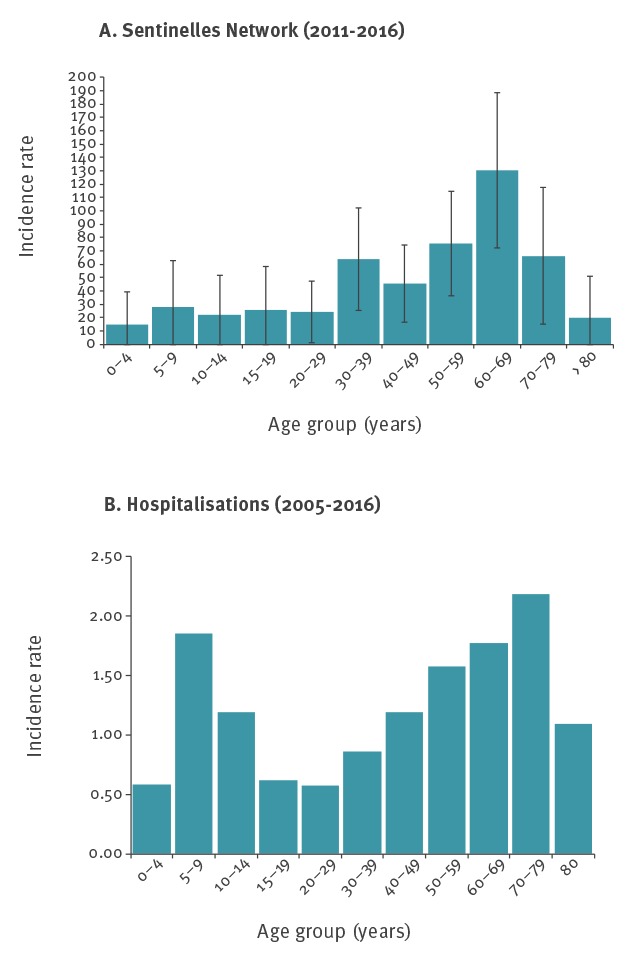
Average yearly rates of Lyme borreliosis by age group in mainland France (A) estimated incidence in general practice, 2011–2016 and (B) hospitalisations, 2005–2016

The highest estimated incidence rate was seen in the 60–69 age group (131 cases per 100,000 (95% CI: 73–188)), followed by the 50–59 age group (76 per 100,000 (95% CI: 36–115)), and the 70–79 age group (66 cases per 100,000 (95% CI: 15–118)) (Figure 1).

Incidence rate is calculated per 100,000 inhabitants.

**Figure 2:**
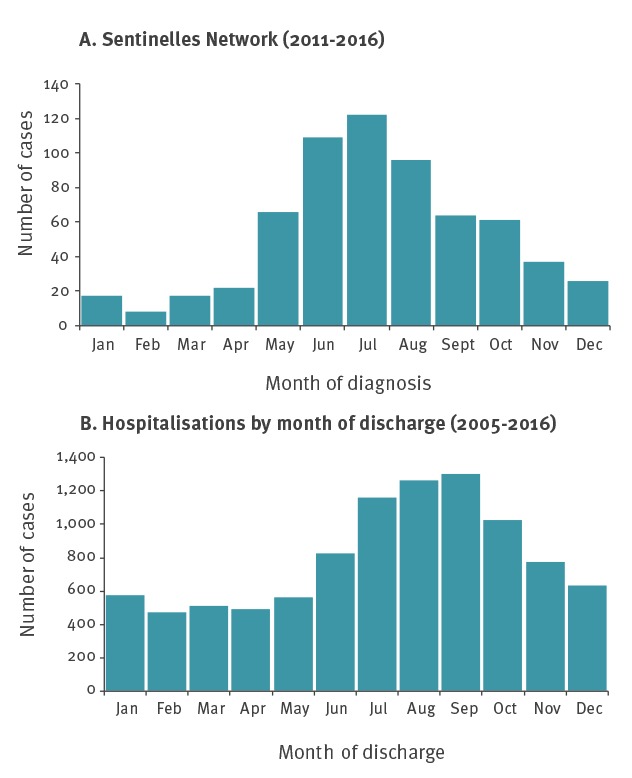
Distribution of Lyme borreliosis cases by month in mainland France (A) in general practice, 2011–2016 and (B) hospitalisations, 2005–2016

LB cases were diagnosed mainly from May to October (n = 518, 78%) over the 2011–16 period, with a peak in July (Figure 2).

**Figure 3:**
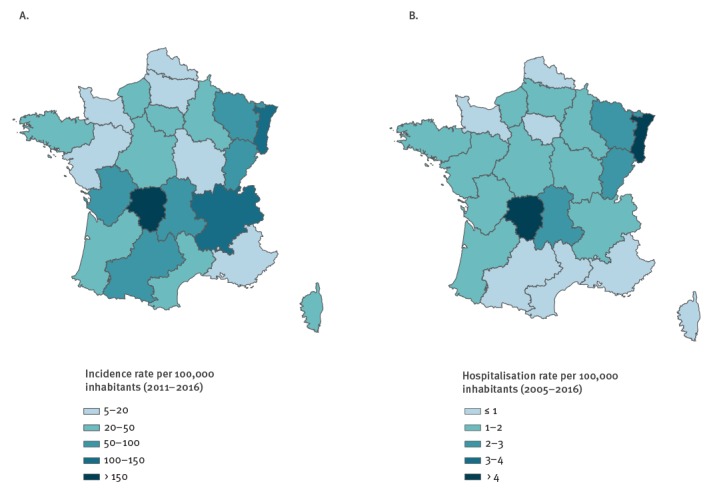
Estimated mean annual regional incidence rates of Lyme borreliosis (A) in general practice, 2011–2016 (B) hospitalisations, 2005–2016

From 2011 to 2016, the regions with the highest average yearly estimated incidence rate per 100,000 inhabitants were Limousin and Alsace, with 239 cases (95% CI: 68–410) and 148 cases (95% CI: 45–251), respectively (Figure 3). The regions with the lowest average yearly incidence rates per 100,000 inhabitants were Pays de la Loire and Provence-Alpes-Côtes-d’Azur with five (95% CI: 0–25) and eight cases (95% CI: 0–20), respectively.

### Lyme borreliosis hospitalisations, 2005–2016

Among the 29,331 discharge reports extracted with a LB code, 11,551 met our inclusion criteria. Accounting for readmissions, 9,594 hospitalised LB cases were identified. Between 2005 and 2016, the mean annual number of hospital stays was 963 (range 846–1,129). The mean annual number of hospitalised cases was 799 (range 649–937). The median number of hospital stays by case was one (range 1–40).

**Figure 4:**
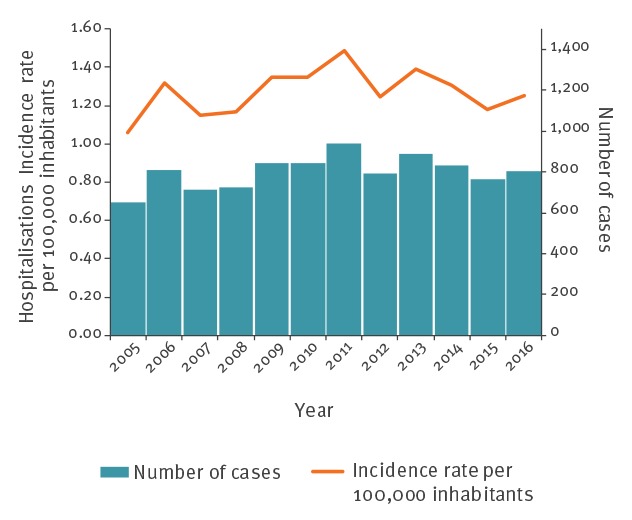
Number of cases hospitalised for Lyme borreliosis and hospitalisation incidence rate per year, French national hospital discharge database^a^, mainland France, 2005–2016

^a^ Programme de Médicalisation des Systèmes d’Information (PMSI).

The average estimated HIR was 1.3 per 100,000 inhabitants per year. The HIR fluctuated from 1.1 per 100 000 inhabitants in 2005 to 1.5 per 100,000 inhabitants in 2011 with no significant trend (p = 0.260) (Figure 4). Regions with the highest incidence rates of LB estimated by the Sentinelles network also had the highest hospitalisation rates (Figure 3). The HIR ranged from 4.2 cases per 100,000 inhabitants per year in Limousin to 0.3 in Corsica and Provence-Alpes-Côte d’Azur.

Reasons for hospitalisation were neurological disorders (n = 4,906; 51%), arthritis (n = 1,250; 13%), cardiac events (n = 639; 7%), ocular disorders (n = 177; 2%) and ACA (n = 231; 2%). For 27% (2,577/9,594) of the hospitalised cases a Lyme disease code (A69.2) was reported in the absence of any other diagnosis.

Of 9,594 cases, 57% were men. Two peaks were observed in the age distribution, in the age groups 5–9 and 70–79 years (Figure 1). The median age was 51 years (range 1–95). Among the 1,805 cases in the 0–15 age group, 62% were hospitalised for neurological disorders compared with 45% (2,058/4,601) in the 15–60 age group and 54% (1,725/3,188) in those aged 60 years and over (p < 0.001). Cases aged 60 years and older were more often hospitalised for cardiac manifestations (n = 320, 11%) than cases 0–15 years (n = 12, 0.7%) and 15–60 years (n = 304, 7%) (p < 0.001). The age and sex distribution are similar over the time period 2005–2016 (data not shown).

The highest numbers of LB hospitalised cases were observed in June–November with a peak every year in August or September (Figure 2). Hospitalised cases with neurological disorders were more often hospitalised during the summer with a peak in September (16%, p < 0.001). For hospitalised cases with cardiac manifestations a peak in August (14%) was observed. However, the incidence of hospitalised cases with cardiac manifestations peaked in August but did not differ significantly (p = 0.758) from the incidence in the other months. Cases with arthritis or ocular disorders were hospitalised evenly throughout the year.

The average length of stay was 6 days (range 0–239) overall, 9.6 days for cases with cardiac disorders, 7.6 days for neurological disorders and 2.7 days for cases with a LB code in the absence of any other associated diagnoses.

### Lyme neuroborreliosis hospitalisations, 2005–2016

Among the 4,906 cases hospitalised with neurological manifestations, 58% were men and the median age was 52 years (range 1–94). The estimated mean annual HIR was 0.6 cases per 100,000 inhabitants ranging from 0.5–0.8 between 2005 and 2011, respectively. Alsace and Limousin were the regions with the highest mean incidence rates with 2.4 cases per 100,000 inhabitants.

Among LNB hospitalised cases, 37% presented facial nerve disorders (including 27% with unilateral facial nerve paralysis), 35% had meningitis, 9% polyneuropathy and 9% encephalitis, myelitis or encephalomyelitis.

**Figure 5:**
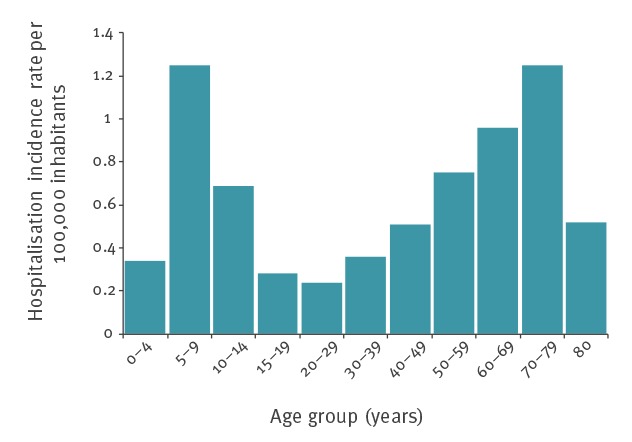
Yearly hospitalisation rates of Lyme neuroborreliosis by age group, mainland France, 2005–2016

HIR of LNB show a bimodal distribution of age with a first peak occurring in children aged 5–9 years old (1.3/100,000 of the age group) and a second peak in adults 70–79 years old (1.3 cases/100,000 of the age group). The proportion of hospitalised cases with LNB differed significantly by age (Figure 5). Among hospitalised cases, those age 5–9 and 70–79 years old were significantly more likely to have had LNB compared with other age groups (odds ratio (OR): 3.02; 95% CI: 2.41–3.78 and OR: 1.94; 95% CI: 1.58–2.39, respectively).

## Discussion

Our study on LB using two complementary data sources, the national Sentinelles GP network (2011–16) and the national hospital discharge database (2005–16), provides an updated overview of the epidemiology of LB in France and documents the trends in incidence of LNB in France. Our results are consistent with those described previously in France [14] and in Europe [6-12,23-27]. 

In France, between 2011 and 2015, the national incidence rates estimated by the Sentinelles network were stable. However, in 2016, an increase in incidence was noted. We found that yearly LB hospitalisation incidence rates fluctuated with no significant trend, whereas in general practice there was a significant increase in LB incidence during 2016 for patients diagnosed with EM. The most frequent clinical manifestation among cases presenting to GPs was EM and given that EM represents the early stage of LB and that antibiotic therapy is effective, this highlights the major role of GPs in secondary prevention of disseminated LB and late manifestations [28]. While there was a predominance of women among the cases consulting to GP, men were predominant among the hospitalised cases. In addition, our data supports the existing evidence that incidence of LNB is higher in children when compared with adults [12].

LB cases who consulted GPs were more often aged 50 years and older and had been bitten by ticks from late spring to early autumn. The numbers of LB cases consulting GPs increased in May and peaked in July, while LB hospital admissions peaked in September. This difference might be due to the delay between infected tick bite(s) and the development of disseminated LB requiring hospitalisation. For individual patients this delay might be difficult to estimate due to recall bias, particularly on the date of the potential infected tick bite(s).

An increase in LB over the last decade has been described in some European countries, but not in others [4]. Regarding countries bordering France, the incidence of LB positive tests reported by the sentinel laboratory network in Belgium (2003–12) and the incidence of GP consultation for EM (90/100,000 inhabitants in 2008–09 and 103/100,000 inhabitants in 2015) were both stable [23,29,30]. In nine states in Germany where LB is mandatory notifiable [27], annual fluctuations in reported numbers of LB cases were observed 2013–17 with no clear increasing or decreasing trend. In Switzerland [26], the incidence of reported LB cases was stable between 2008 and 2011 (mean annual incidence 131/100,000 inhabitants). In the Netherlands a continuous increase in incidence of GP consultations for EM was observed 1994–2014 when the incidence stabilised at 140/100,000 inhabitants [7,24]. In parallel, a decrease in tick bite consultations was observed in 2014, that may reflect the impact of public health education interventions (in particular body checking and prompt tick removal). No significant change in *Ixodes ricinus* abundance was reported in the Netherlands 2009–14 [24].

In European countries, surveillance of LB is based on different case definitions and surveillance systems: voluntary/compulsory reporting, laboratory reporting vs physician reporting, or hospital diagnoses [4,5]. The use of a common case definition developed by EUCALB is a first step towards harmonisation. Since 2011, the EUCALB case definition includes a criterion on the size of EM (greater or equal to 5 cm) and this definition can be used when comparing data from different countries.

However, comparisons between countries must be interpreted with caution. Heterogeneity among surveillance systems, difference in how healthcare is accessed and varying practices with regard to diagnostic investigations can all impact the estimates. These surveillance artefacts should be distinguished from differences in the genuine incidence rates that are conditioned by geographical, environmental (land use and density of animal reservoirs) and climatic factors, as well as the heterogeneous distribution of *Borrelia burgdorferi *sensu lato species, among others.

Our results suggest that mainland France has an estimated LB incidence rate similar to that of bordering countries with comparable environmental conditions (Germany, Belgium and Switzerland) but higher than countries such as Spain and Italy where environmental conditions do not favour the presence of *Ixodes ticks* [4,31].

The increase in incidence of LB cases in 2016 in general practice was not observed in hospitals neither at the national level nor in high incidence regions. This difference in trends could be explained by several hypotheses. In 2016, following the launch of the national plan against Lyme disease and tick-borne diseases [32], information and training activities were conducted for the general public and health professionals to increase awareness, specifically in regard to better detection of tick bites and skin lesions, possibly leading to increased consultations in general medicine. In addition, the media have largely covered this subject. The increase in incidence observed in 2016 by the Sentinelles network may therefore be the result of a surveillance bias due to better case detection and the incidence might have been underestimated in previous years. It is also possible that the incidence of EM actually increased in 2016, but because of appropriate care at the primary care level, this increase in incidence did not translate in an increase in the number of hospitalised cases. Hypothetically, this increase might be also due to special climate conditions, such as a mild winter followed by a warm and wet summer, as was observed in Sweden between 1999 and 2000 [10]. However, according to the 2016 weather report by Météo France, climatic conditions were not particularly favourable for the tick activity. Indeed, rainfall between July and September 2016 was one of the lowest in this period since 1959 [33].

Only a few European countries have published hospital discharge data related to LB. In Germany, a study analysing a large nationwide health insurance database, estimated the yearly LB hospitalisation incidence rate for the period 2008–11 to be nine per 100,000 inhabitants [34]. In Finland a study of the national hospital database estimated this incidence in 2014 at 19 per 100,000 [25]. These estimations are higher than the estimate in France (1.3/100,000). In the German and Finnish studies, the case definition was based only on the ICD-10 code A69.2. In our study, the hospitalisation incidence rate of patients with the A69.2 code (without other criteria), was 2.8 cases per 100,000 inhabitants. In Sweden [35], the incidence of LNB determined using the Swedish hospital discharge database, was estimated at 5.2 cases per 100,000 inhabitants, higher than in France (0.6/100,000 inhabitants).

The highest estimated incidence rate and the highest hospitalisation rate over the study period were in eastern and central regions of mainland France. It should be noted that these estimates were based on place of residence or hospitalisation and not place of infection.

The vector, the tick *Ixodes ricinus* is present in most parts of mainland France, except above 1,200 m and in the dry Mediterranean areas [36]. *Ixodes ricinus* ticks, the primary vector in Europe, are usually found in vegetation types with deciduous or mixed woodland that maintain high humidity (requiring a relative humidity of at least 80%) and in areas of moderate to high rainfall, such as in eastern and central regions of mainland France [4,36-38]. Since infection is correlated with tick abundance and human to tick exposure, this variability in incidence rates could be explained by differences in geographical and climate characteristics, in types of exposure (recreational and occupational exposure to ticks and outdoor activities) and presence of competent reservoir hosts. Tick nymphs are mainly responsible for transmitting *Borrelia* to humans and quest most actively from spring to autumn [4,36,37]. Diagnoses of acute LB peak in summer in many northern and central countries of Europe [4]. These findings were also confirmed in our study with higher incidences of LB from July to September.

In the absence of further information about distribution and density of ticks in France, surveillance data can be used to guide future studies such as research studies on ecology of the vector and its reservoir or the prevalence of infection in ticks by geographical areas and to target public health actions such as health communication campaigns to the most affected populations.

Importantly, over the study period, there was no change in the validation protocol, case definitions, SGPs participation and methods used to estimate regional and national incidences, strengthening the reliability of our results in terms of trends and LB incidence estimates. Incidences estimated by the Sentinelles network account only for patients consulting GPs. If it is assumed that the proportion of cases not presenting to GP remained stable over the study period, this method is suitable to follow the trend in incidence of LB. EM is the most discriminating sign enabling a reliable clinical diagnosis at the primary level care and is therefore a key indicator for LB surveillance [39]. These data permit to target public health communication about preventive measures for the general population including prevention of tick bites and LB and elaborate guidelines on the prevention and diagnosis for health professionals [40].

### Limitations

There are important limitations in using data from the national hospital discharge database, including diagnosis and coding errors, involuntary omissions and reporting of pre-existing conditions not related to the stay, as well as lack of information about clinical symptoms and laboratory diagnostic results which could help to validate the diagnosis. Therefore, it is possible that we overestimated the hospitalisation rate when LB was coded but not directly related to the cause of hospitalisation. It is also possible that diagnoses of LB are undercoded or underdiagnosed and so the incidence may be underestimated. These biases are inherent to the use of the hospital discharge database [20] since this database was initially created as a tool for resource allocation [20].

The algorithm of codes we used to define LB, developed by a team of clinicians and epidemiologists, also has its limitations. We can overestimate the number of LB cases if the retained case definitions have low specificity. Because an earlier French study of hospital files estimated that the positive predictive value of a case definition based on the presence of at least one specific ICD Lyme code (A69.2 or M01.2) was only 65%, we decided to have a more specific case definition. By doing so, we aimed to reduce the background noise of inclusion of ‘false positive cases’ which could hinder the interpretation of the trends. However, we may have still included non-confirmed cases but it is also likely that our case definitions lack sensitivity and that we underestimate the true incidence. Existing guidelines for the diagnosis of LNB in Europe are based on clinical symptoms and laboratory analysis, particularly intrathecal specific antibody production [39,41]. It will be important to study in more depth how ICD Codes compare to laboratory data and to further validate the algorithms.

Meanwhile, in spite of the limitations, the hospitalisation database provides useful data and allows for monitoring of trends over time. In addition, it can be used to determine seasonality, high-risk regions and characteristics of hospitalised patients. The method and the database used are stable which are essential attributes required for trend analysis. Furthermore, the PMSI database has coding rules to minimise errors and variations between institutes and is a comprehensive national system providing opportunities to implement national level studies [42].

### Conclusion

The combination of a sentinel network of GPs and the hospitalisation discharge database permits monitoring of two key indicators: EM and LNB and therefore provides a more comprehensive understanding of the epidemiology of LB in France. Furthermore, these data sources provide information at a regional level allowing the analysis of the geographical distribution and potential expansion of LB across the country.

Public health strategies should focus on age groups and regions with a high incidence of LB (particularly during the months with the highest incidence) and should emphasise prevention measures such as regular tick checks after exposure and prompt removal to avoid infection.
